# Multi-GNSS PPP-RTK: From Large- to Small-Scale Networks

**DOI:** 10.3390/s18041078

**Published:** 2018-04-03

**Authors:** Nandakumaran Nadarajah, Amir Khodabandeh, Kan Wang, Mazher Choudhury, Peter J. G. Teunissen

**Affiliations:** 1 GNSS Research Centre, Department of Spatial Sciences, Curtin University, Perth, WA 6845, Australia; N.Nadarajah@curtin.edu.au (N.N.); amir.khodabandeh@curtin.edu.au (A.K.); kan.wang@curtin.edu.au (K.W.); mohammad.choudhury@curtin.edu.au (M.C.); 2 Department of Geoscience and Remote Sensing, Delft University of Technology, 2628 CN, Delft, The Netherlands

**Keywords:** Global Navigation Satellite Systems (GNSS), PPP-RTK network and user, network in-loop, carrier phase ambiguity resolution, ionosphere weighted model

## Abstract

Precise point positioning (PPP) and its integer ambiguity resolution-enabled variant, PPP-RTK (real-time kinematic), can benefit enormously from the integration of multiple global navigation satellite systems (GNSS). In such a multi-GNSS landscape, the positioning convergence time is expected to be reduced considerably as compared to the one obtained by a single-GNSS setup. It is therefore the goal of the present contribution to provide numerical insights into the role taken by the multi-GNSS integration in delivering fast and high-precision positioning solutions (sub-decimeter and centimeter levels) using PPP-RTK. To that end, we employ the Curtin PPP-RTK platform and process data-sets of GPS, BeiDou Navigation Satellite System (BDS) and Galileo in stand-alone and combined forms. The data-sets are collected by various receiver types, ranging from high-end multi-frequency geodetic receivers to low-cost single-frequency mass-market receivers. The corresponding stations form a large-scale (Australia-wide) network as well as a small-scale network with inter-station distances less than 30 km. In case of the Australia-wide GPS-only ambiguity-float setup, 90% of the horizontal positioning errors (kinematic mode) are shown to become less than five centimeters after 103 min. The stated required time is reduced to 66 min for the corresponding GPS + BDS + Galieo setup. The time is further reduced to 15 min by applying single-receiver ambiguity resolution. The outcomes are supported by the positioning results of the small-scale network.

## 1. Introduction

Integer ambiguity resolution-enabled precise point positioning, PPP-RTK, enables single-receiver users to recover the integerness of their carrier-phase ambiguities, thereby reducing the positioning convergence time as compared to that of PPP [1,2]. Apart from the satellite clocks, the PPP-RTK network platform also provides users with the satellite phase biases and (optionally) the ionospheric corrections for fast integer ambiguity resolution [3,4]. Compared to the standard PPP technique, which normally requires long convergence time from tens of minutes to hours to reach cm-level accuracy [5,6,7], the resolved integer ambiguities in PPP-RTK lead to shorter convergence time in user positioning results. Using 30–s single-frequency GPS data, the ambiguities can be resolved within several minutes with the rover positioning precision reduced to mm- and cm-level in the horizontal and vertical directions [3].

To achieve fast integer ambiguity resolution and high accuracy of the rover positionng results, studies were performed using different PPP-RTK implementations [2,8,9,10,11,12]. A detailed review of the different mechanisations of PPP-RTK and their intricacies are given in [13]. In this study, observations are processed using the Curtin PPP-RTK Software [14,15] at the undifferenced and uncombined level [14]. This does not only lead to convenience in extending the number of the frequencies and satellite systems involved, but also provides possibilities to apply rigorous spatial and temporal dynamic models on parameters like clocks, hardware biases and ionospheric delays [16]. To remove the rank deficiencies in the design matrix, estimable parameters are formed based on the S-system theory [17,18]. With the estimated satellite clocks, satellite phase biases and optionally the ionospheric delays provided to the users, the user platform performs positioning in either static or kinematic mode and distinguishes between the ‘ionosphere-float’ and ‘ionosphere-weighted’ scenarios without and with the ionosphere information delivered by the network platform.

Using the Curtin PPP-RTK software [14] has provided an overview of the PPP-RTK user positioning results using the dual-frequency GPS data from an Australia-wide network in an ionosphere-float scenario. An outlook was also given for multi-frequency multi-GNSS PPP-RTK and the ionosphere-weighted scenario. As a realization of this outlook, in this contribution, user positioning results are computed using corrections provided by an Australian-wide network and a small-scale network of 30 km based on multi-GNSS data of GPS, BDS and Galileo in comparison with those obtained by the corresponding GPS-only data. Results based on single-frequency multi-GNSS low-cost network are also analysed for PPP-RTK and in-loop users. Thus, we describe and demonstrate in this contribution the versatility and generality of our modelling approach as it is mechanized in the Curtin multi-GNSS network and user platform. To cover the broad range of applications, from high-end to low-end users, we present our analyses for different classes, from large-scale to small-scale to single-frequency PPP-RTK with cheap receivers. We thereby also carefully describe and analyse the various differences that occur in the estimability of the parameters. This important point is usually not explicitly addressed in the literature but is crucial for a proper understanding of the physical parameters that one is actually estimating. In the next section, the paper starts with an introduction of the processing strategy and the interpretation of the estimable parameters in the Curtin PPP-RTK platform. After that, the multi-GNSS user positioning results based on an Australia-wide network, a small-scale network and a low-cost network are analysed with the convergence curves in different scenarios. The last section concludes the results presented in this contribution.

## 2. GNSS-Derived Estimable Parameters Output by Curtin’s PPP-RTK Platform

In this section, the GNSS measurement and dynamic models on which Curtin’s PPP-RTK platform is based are briefly reviewed. We commence with the main GNSS observations underlying the stated models, i.e., the *undifferenced* (UD) carrier-phase and pseudo-range (code) data collected on multiple frequencies.

### 2.1. Undifferenced Phase and Code Observation Equations

Let the observed-minus-computed carrier-phase and code observations of receiver *r* (r=1,⋯,n), tracked by satellite *s* (s=1,⋯,m), be denoted by $\Delta \phi_{r,j}^{s}$ and $\Delta p_{r,j}^{s}$, respectively. The subscript *j* (j=1,⋯,f) indicates the frequency on which the observations are collected. The corresponding observation equations read [19]

(1) $$\begin{array}{ccc} \Delta \phi_{r,j}^{s} & = & \Delta \rho_{r}^{s}+g_{r}^{s}\;\tau_{r}-\mu_{j}\;\iota_{r}^{s}+dt_{r}-dt^{s}+\delta_{r,j}-\delta_{,j}^{s}+\lambda_{j}a_{r,j}^{1s},\;\\ \Delta p_{r,j}^{s} & = & \Delta \rho_{r}^{s}+g_{r}^{s}\;\tau_{r}+\mu_{j}\;\iota_{r}^{s}+dt_{r}-dt^{s}+d_{r,j}-d_{,j}^{s}, \end{array}$$

where $\Delta \rho_{r}^{s}=c_{r}^{sT}\Delta x_{r}$ denotes the ‘increment’ of the geometric range between receiver *r* and satellite *s*, containing the receiver’s position increment $\Delta x_{r}$. Thus, the 3×1 vector $c_{r}^{s}$ represents the receiver-to-satellite unit direction vector. While the hydrostatic components of the zenith tropospheric delays (ZTDs) are a priori corrected using the Saastamoinen model, the wet components, denoted by $\tau_{r}$, are treated as unknowns and linked to the observations through the mapping functions $g_{r}^{s}$. The first-order slant ionospheric delays, experienced on the first frequency j=1, are denoted by $\iota_{r}^{s}$. Thus, the corresponding coefficient is given as $\mu_{j}={\left(\lambda_{j}/\lambda_{1}\right)}^{2}$, with $\lambda_{j}$ being the carrier-phase wavelength on frequency *j*. The receiver and satellite clock parameters are denoted as $dt_{r}$ and $dt^{s}$, respectively. The frequency-dependent receiver and satellite code biases are denoted by $d_{r,j}$ and $d_{,j}^{s}$, respectively. Likewise, $\delta_{r,j}$ and $\delta_{,j}^{s}$, respectively, denote the receiver and satellite phase biases. The integer-valued ambiguities are denoted as $a_{r,j}^{s}$. All quantities are expressed in units of range, except the ambiguities $a_{r,j}^{s}$, which are given in cycles. The precise satellite orbits are assumed to be included in $\Delta \phi_{r,j}^{s}$ and $\Delta p_{r,j}^{s}$.

### 2.2. Dynamic Models: Temporal Behaviour of the Parameters

The UD formulation (1) represents a *rank-deficient* system of observation equations, that is, not all of the parameters involved in (1) are estimable, only combinations of them. A number of parameters, equal to the rank-deficiency, must therefore be chosen as the system’s S-basis so as to form a set of minimum constraints that recovers the system of equations to one of full rank [17,18]. From an algorithmic point of view, this means that one has to remove those columns of the model’s design matrix corresponding to the S-basis parameters [16]. The number of the parameters required to be chosen as S-basis is prone to be affected by any assumption placed on the underlying dynamic model as well as on the measurement model. For instance, the receivers may collect data on only *one* frequency or on *multiple* frequencies. The temporal behavior of the parameters, involved in (1), may be *modelled* or some (all) of the parameters may be assumed to be *unlinked in time*. Each of the stated scenarios changes the number of S-basis parameters. Clearly, by removing the corresponding columns of the model’s design matrix, one would not estimate the remaining parameters in an unbiased manner. In other words, the interpretation of the remaining parameters does change. For instance, consider the case where all the parameters, except the clocks (and ionospheric delays), are assumed linked in time. The interpretations of the remaining parameters, referred to as the *estimable* parameters, are given in Table 1. In the table, the estimable parameters are distinguished from their original counterparts by the $\tilde{.}$-symbol. As shown, the estimable slant ionospheric delays at epoch *i*, i.e., ${\tilde{\iota }}_{r}^{s}\left(i\right)$, do not represent their original versions, namely, the unbiased slant ionospheric delays $\iota_{r}^{s}\left(i\right)$. Instead, they represent *biased* slant ionospheric delays that are lumped with the (scaled) differential code biases (DCBs) $d_{r,GF}\left(1\right)$ and $d_{,GF}^{s}\left(1\right)$ at the first epoch i=1. In this contribution, we present results under the following two different dynamic models:

- Time-unlinked clocks: While both the receiver and satellite clocks are treated unlinked in time, the temporal behaviour of the remaining parameters (except the ambiguities) are modeled as a ‘random-walk’ process. The ambiguities are assumed constant in time, unless slips occur (cf. Section 3.1).
- Time-linked satellite clocks: While the receiver clocks are assumed unlinked in time, the temporal behaviour of the satellite clocks is modeled by a constant-velocity dynamic model (i.e., the 2-state clock-plus-drift model) [15] (cf. Section 3.3).

### 2.3. Measurement Models

Next to the dynamic models that concern the temporal behaviour of the parameters, any assumption underlying the measurement model can also affect the rank-deficiencies involved in Equation (1). For instance, if one would parametrize the slant ionospheric delays $\iota_{r}^{s}$ into a fewer number of unknown parameters using, e.g., the single-layer models [20], the interpretation of the estimable parameters in Table 1 changes. The following measurement models are considered:

*– From small- to large-scale networks*. Consider the case where the inter-station distances between the network receivers are so short such that all receivers view satellite *s* from almost the *same* direction angle. Thus, $c_{r}^{s}\approx c_{1}^{s}$ and $g_{r}^{s}\approx g_{1}^{s}$ (r=2,…,n). As a consequence, the GNSS data cannot distinguish between the tropospheric delays $g_{1}^{s}\tau_{1}$ (or the geometric delays $\Delta \rho_{1}^{s}$) and the delays caused by the satellite clocks $dt^{s}$ in Equation (1). To tackle such near rank-deficiency between the ZTDs $\tau_{r}$ (position increments $\Delta x_{r}$) and the satellite clocks $dt^{s}$, one has to therefore choose the reference ZTD $\tau_{1}\left(1\right)$ (reference position $\Delta x_{1}\left(1\right)$) as an additional S-basis parameter [16]. As shown in Table 1, the estimable ZTDs ${\tilde{\tau }}_{r}$ do not represent the unbiased ZTDs $\tau_{r}$, but ZTDs relative to the reference ZTD $\tau_{1}\left(1\right)$ at the first epoch i=1. Similarly, the estimable satellite clocks $d{\tilde{t}}^{s}$ are further biased by the terms $g_{1}^{s}\tau_{1}\left(1\right)$ and $c_{1}^{sT}\Delta x_{1}\left(1\right)$. In case of large-scale networks, however, the stated rank-deficiency is absent. Numerical results concerning both the large-scale and small-scale networks are presented in Section 3 and Section 4, respectively.

*– Fully- and partially-known receivers’ positions*. In the system of observation Equation (1), the positions of the network receivers are assumed unknown. Note, however, that the positions of some (or all) of the network receivers can be a priori known. For instance, in case of continuously operating reference station (CORS) networks, the positions of all the network receivers are assumed known. Next to the CORS network setup, we also consider the case where the positions of some of the receivers are fully unknown. This is particularly useful when the network is extended by newly-established stations whose positions are required to be determined.

*– Ionosphere-float and -weighted models*. Due to the spatial correlation of the ionosphere, the slant ionospheric delays experienced by nearby receivers from a given satellite are almost identical. One can therefore make use of this property and augment the observation Equation (1) with the following extra observation Equation [21]:

(2) $$d\iota_{r}^{s}=\iota_{r}^{s}-\iota_{1}^{s},\;r=2,\ldots ,n$$

in which $d\iota_{r}^{s}$ denote the ionospheric pseudo-observations taking *zero* samples values. The smaller the distance between receiver *r* and r=1, the better the approximation $0\approx (\iota_{r}^{s}-\iota_{1}^{s})$ becomes. Therefore, one can assign weights to the pseudo-observations $d\iota_{r}^{s}$ such that the weights increase when the inter-station distances decrease. Examples of such weights are presented in, e.g., (Appendix A, [15]). When the GNSS observation Equation (1) is augmented with Equation (2), we refer to the model as the *ionosphere-weighted* model. In the absence of the extra observation Equation (2), the underlying model is referred to as the *ionosphere-float* model. Both of these models are considered in the following.

## 3. PPP-RTK Results: A Large-Scale Network

**Figure 1:**
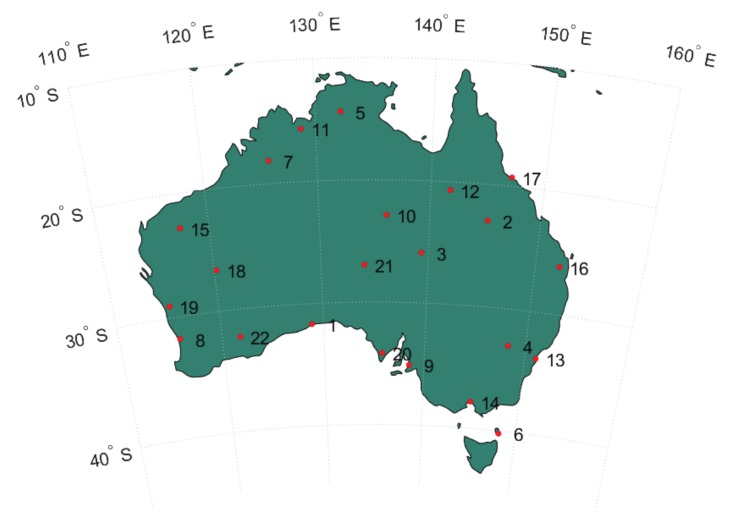
A network of 22 multi-GNSS continuous operating reference stations (red dots) over Australia. The stations are equipped with various receiver types including Trimble NetR9, Septentrio PolRx5, Septentrio PolaRx4TR, Septentrio PolaRx4TR, Septentrio PolaRxS, and Leica GR30.

This section presents PPP-RTK network and user results based on processing of multi-GNSS data from Australia-wide network and user stations tracking at least GPS, BDS, and Galileo satellites. As depicted in Figure 1, the network is formed by 22 multi-GNSS continuous operating reference stations consisting of various receiver types including Trimble NetR9 (Sunnyvale, CA, USA), Septentrio PolRx5 (Leuven, Belgium), Septentrio PolaRx4TR, Septentrio PolaRx4TR, Septentrio PolaRxS, and Leica GR30 (St. Gallen, Switzerland). Results discussed in the following sections are based on data collected from these network stations and 50 user stations across Australia for ten days across three months (every seventh day) starting from day of year 340 of year 2016 with 30 s sampling interval. Station 1 (UCLA, Eucla in Western Australia) is arbitrarily selected as the reference station for PPP-RTK network processing.

### 3.1. Network Results

Multi-GNSS network data was processed with Curtin’s PPP-RTK Network platform. Satellite positions were determined using precise IGS (International GNSS Service) Multi-GNSS Experiment (IGS-MGEX) orbits [22,23,24] while station coordinates were extracted from Geoscience Australia’s Solution Independent Exchange format (SINEX) files. The estimable satellite clocks of multi-GNSS observables were aligned to respective reference observables using IGS-MGEX satellite DCB files. Stochastic, dynamic, and ambiguity resolution parameter settings for network processing are summarized in Table 2.

**Figure 2:**
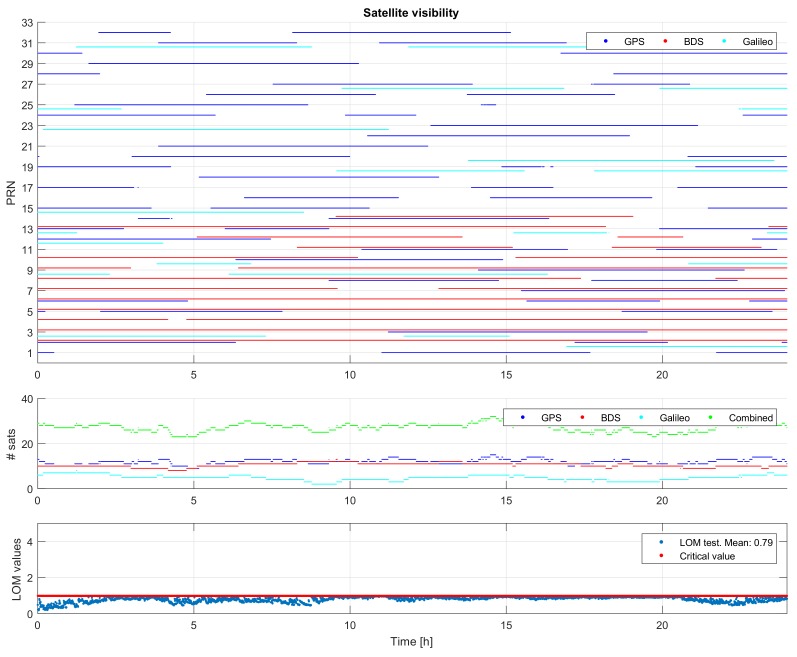
Satellite visibility, number of satellites, and Local Overall Model (LOM) test outcomes.

Figure 2 depicts satellite visibility and Local Overall Model (LOM) test statistics [25]. The top panel indicates the time period(s) for which any given satellite of GPS, BDS, and Galileo is in common view of at least two stations. The middle panel shows the number of satellites visible above 10 degrees for individual systems as well as combined systems. As of the time of data collection, the number of satellites of combined (GPS, BDS, and Galileo) systems varies from 23 to 32. The bottom panel shows the time-series of LOM test statistics, along with their critical values. These LOM test values are obtained in the Detection step, which is the first step of the Main Detection-Identification-Adaptation (DIA) procedure. The purpose of the DIA procedure, implemented in Curtin PPP-RTK platform, is to identify mis-modeled effects (e.g., phase slips and code outliers) and adapt the model accordingly. After identifying mis-modeled effects and successful model’s adaptation, all LOM values are found to be less than their critical values during the day, indicating the validity of the network model used.

**Figure 3:**
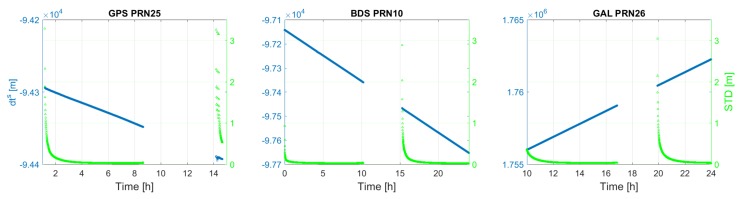
GPS, BDS, Galileo satellite clock error.

**Figure 4:**
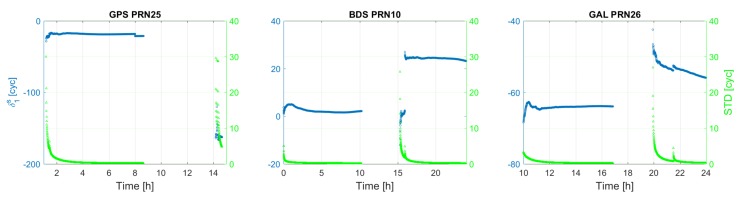
GPS-L1, BDS-B1, Galileo-E1 satellite phase biases.

**Figure 5:**
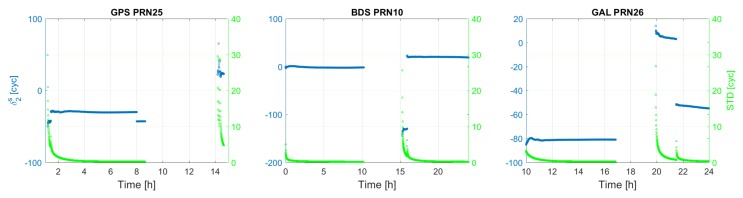
GPS-L2, BDS-B2, Galileo-E5a satellite phase biases.

Figure 3 shows the time series of the satellite clock estimates ($\delta {\tilde{t}}^{s}$), together with their formal standard deviations for an arbitrary satellite per system. It usually takes about 2 h to have satellite clock estimates with formal standard deviations lower than 2 cm. It can be seen that, for each satellite shown, its clock estimates behave almost linearly as a function of time, which is explored for satellite clock modeling in Section 3.3. Figure 4 and Figure 5 depict the first and second frequency satellite phase bias estimates (${\tilde{\delta }}_{,j}^{s}$) together with their formal standard deviations. Apart from the jumps, satellite phase biases are quite stable during a satellite’s pass. The formal precision of the satellite phase biases reaches a stable level of about 0.15 cycle after 3 h. The jumps that are visible in satellite phase bias estimates are caused by a change in pivot ambiguity.

### 3.2. User Results

With availability of network-derived satellite clock and satellite phase bias products from network processing above, the convergence behavior of user position is analyzed next using multi-GNSS data from 50 CORS receivers evenly distributed across Australia. The associated parameter settings for user processing were identical to that of network processing above, except that user position was assumed to be unknown and unlinked in time. To get enough samples for generating reliable convergence curves, user processing was repeated every hour and across ten days resulting in 3800 data windows. The earliest user processing window each day started an hour after the initialization of respective network processing allowing network derived products to converge. The user processing was repeated for four different GNSS combinations: GPS-only, GPS + BDS, GPS + Galileo, GPS + BDS + Galileo.

**Figure 6:**
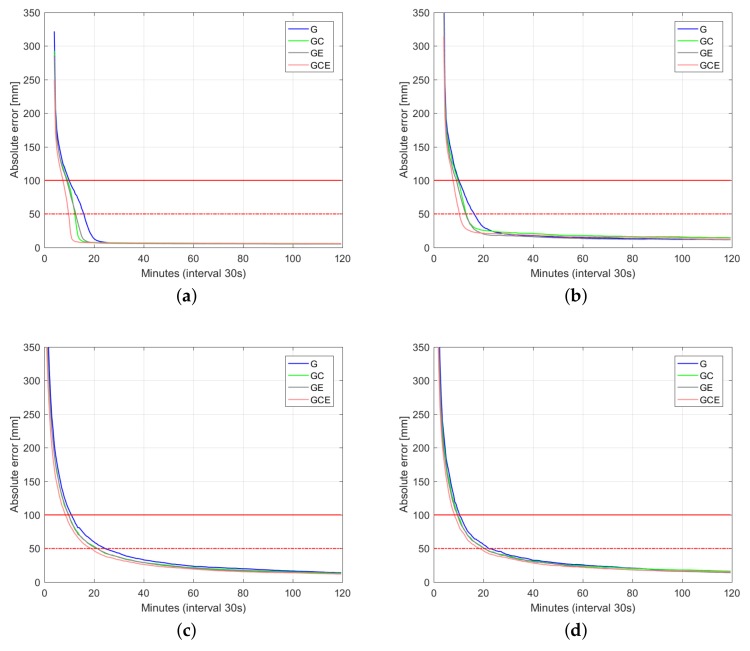
Convergence behavior (50% of samples) of the horizontal radial position error and up component error based on processing 3800 data windows of multi-GNSS (GPS, BDS, Galileo), dual-frequency (L1-L2, B1-B2, E1-E5a) Australia-wide network data. (**a**) horizontal radial (fixed); (**b**) up component (fixed); (**c**) horizontal radial (float); (**d**) up component (float).

**Figure 7:**
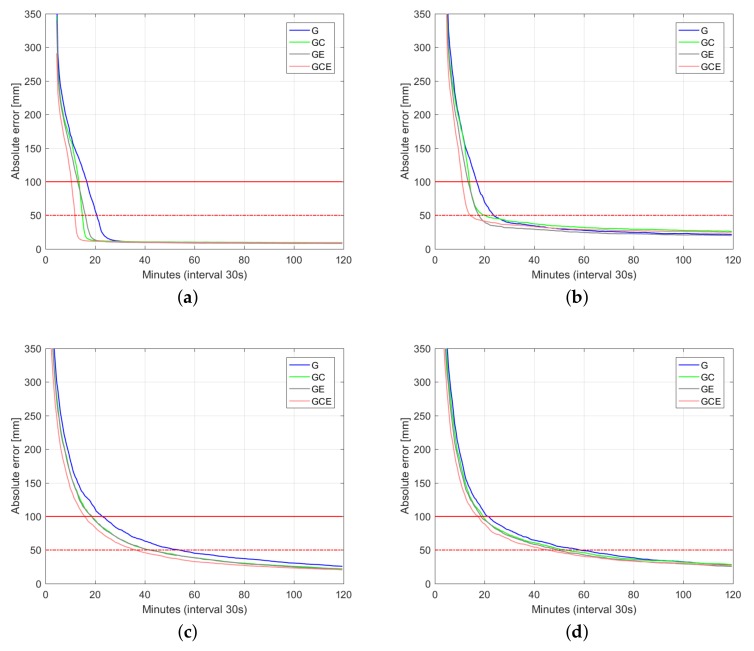
Convergence behavior (75% of samples) of the horizontal radial position error and up component error based on processing 3800 data windows of multi-GNSS (GPS, BDS, Galileo), dual-frequency (L1-L2, B1-B2, E1-E5a) Australia-wide network data. (**a**) horizontal radial (fixed); (**b**) up component (fixed); (**c**) horizontal radial (float); (**d**) up component (float).

**Figure 8:**
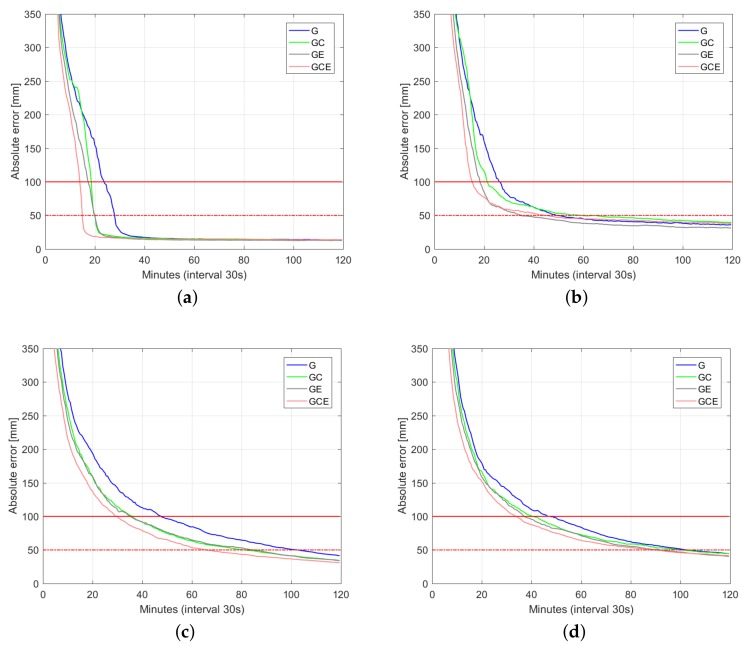
Convergence behavior (90% of samples) of the horizontal radial position error and up component error based on processing 3800 data windows of multi-GNSS (GPS, BDS, Galileo), dual-frequency (L1-L2, B1-B2, E1-E5a) Australia-wide network data. (**a**) horizontal radial (fixed); (**b**) up component (fixed); (**c**) horizontal radial (float); (**d**) up component (float).

Figure 6, Figure 7 and Figure 8 depict 50%, 75%, and 90% (horizontal radial and up component) convergence curves respectively, while Table 3, Table 4 and Table 5 summarize corresponding convergence time for 1 dm and 5 cm (in brackets). The figures on the first row correspond to ambiguity fixed solution, while the figures on the second row are based on ambiguity float solution. As in precise point positioning (PPP), with time-constant ambiguities, the precision of ambiguity float solution is gradually driven by precise phase observations. With partial fixing option for ambiguity resolution, once a big enough number of ambiguities attained prescribed precision, the ambiguity resolved solution reaches convergence criteria (1 dm defined by red solid line and 5 cm defined by red dashed line) faster than the ambiguity float solution, demonstrating the benefit of ambiguity resolution. Integrating multiple systems increases the redundancy of the underlying model, and hence improves the convergence performance. Even though the number of visible Galileo satellites is less than that of BDS (Figure 2), GPS + Galileo processing is almost performing equally to GPS + BDS in terms of user position convergence.

### 3.3. Impact of Latency and Satellite Clock Modelling

With the dynamic satellite clock model incorporated in the network Kalman filter (Table 6), the satellite clock rate $d{\dot{\tilde{t}}}^{s}$ is able to be estimated at each epoch [15]. In case of latency of the network products, denoted by Δi, the estimable satellite phase biases and satellite clocks can be time-predicted with

(3) $$\begin{array}{ccc} d{\overset{ˇ}{\tilde{t}}}^{s}\left(i+\Delta i\right) & = & d{\hat{\tilde{t}}}^{s}\left(i\right)+\left(\Delta i\right)\;d{\hat{\dot{\tilde{t}}}}^{s}\left(i\right), \end{array}$$

(4) $$\begin{array}{ccc} {\overset{ˇ}{\tilde{\delta }}}_{,j}^{s}\left(i+\Delta i\right) & = & {\hat{\tilde{\delta }}}_{,j}^{s}\left(i\right). \end{array}$$

In this study, the satellite clocks are modeled with white frequency noise (WFN) with the process noise set to be 0.001 m/$\sqrt{s}$ [15]. With 1 Hz GPS dual-frequency data of the Australia wide network processed from 13:00:00 to 14:59:59 in GPS Time (GPST) on 12 October 2017, the network products were estimated without latency and predicted with latencies of 10 and 15 s. The starting time of the user processing varies from 13:40:00 to 14:00:00 with a time shift of 1 min and continues for 1 h for each station. Using 51 user stations, more than 1000 data samples with 1 h coordinate time series were generated and used for computing the convergence curves for different latencies in ambiguity-fixed and -float cases.

**Figure 9:**
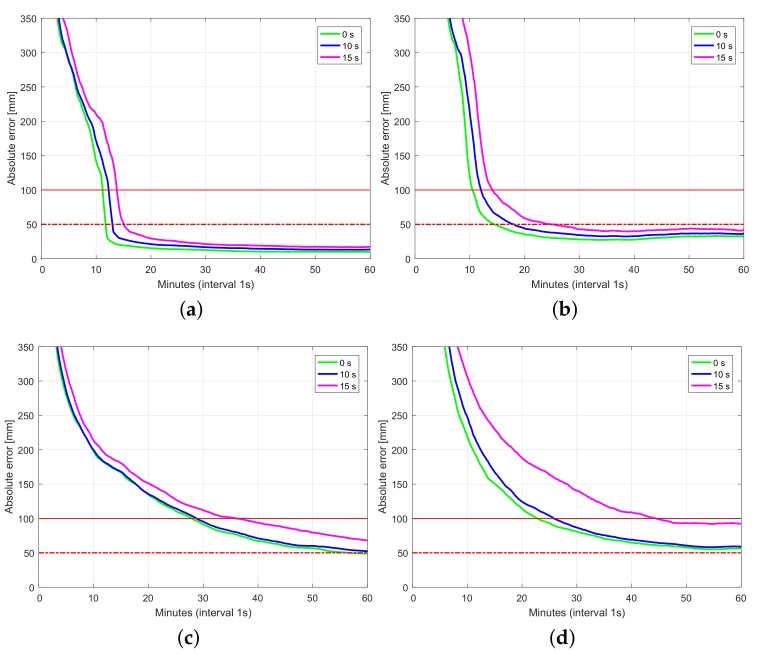
Convergence behavior (75% of samples) of the horizontal radial position error and up component error in (**a**,**b**) ambiguity-fixed and (**c**,**d**) -float cases. The network products were estimated without latency (green lines) and predicted with latencies of 10 s (blue lines) and 15 s (magenta lines). 1 Hz GPS dual-frequency data on L1 and L2 were used for the processing. (**a**) horizontal radial (fixed); (**b**) up component (fixed); (**c**) horizontal radial (float); (**d**) up component (float).

Figure 9 shows the 75% convergence curves of the horizontal radial components and the absolute up components of the user coordinates deviated from the ground truth. The network products were estimated without latency (see the green lines in Figure 9) and predicted with latencies of 10 and 15 s (see the blue and magenta lines in Figure 9). The red lines mark the *y*-values of 5 cm and 1 dm. We see that the convergence time to 1 dm increases with the increasing latencies. For ambiguity-float case, more time is required to reduce the coordinate increments to 1 dm compared to the ambiguity-fixed case. With the ambiguities fixed and the network products estimated without latency, it takes around 10 min to let the horizontal and up coordinate increments reduce to below 1 dm in 75% of the cases (see also the green lines in Figure 9a,b). As the latency of the network products increases, more time is needed. With a latency of 15 s (see also the magenta lines in Figure 9a,b), it takes around 15 min to reduce the coordinate increments to below 1 dm. In ambiguity-float case, more time is required for the coordinate convergence. As shown by the green lines in Figure 9c,d, it takes around 20 to 30 min to reduce the coordinates to 1 dm without latency of the network products. With a latency of 15 s (see also the magenta lines in Figure 9c,d), it takes around 35 to 45 min to reach an coordinate increment of 1 dm.

## 4. PPP-RTK Results: A Small-Scale Network

**Figure 10:**
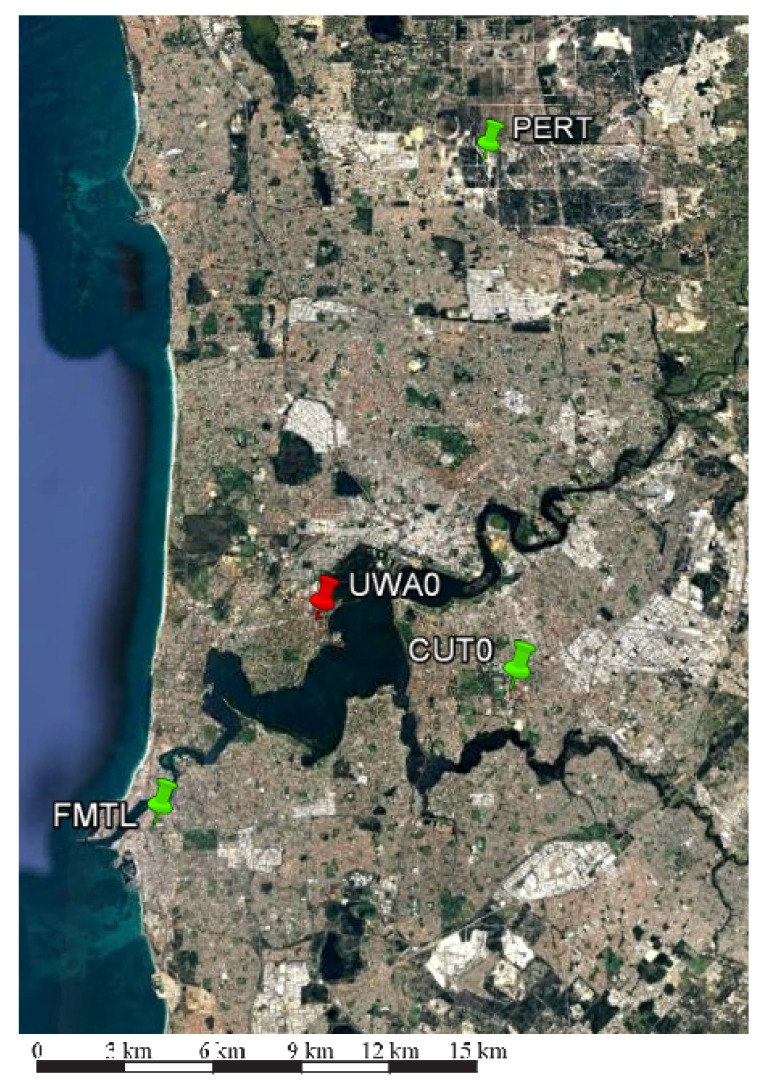
Multi-GNSS, small-scale network (30 km) (Map data @ 2018 Google, Data SIO, NOAA, U.S. Navy. NGA. GEBCO) [26].

This section presents user positioning performance results of a single user (UWA0) in a small-scale network (30 km) formed by three stations (shown in green in Figure 10). All three network stations are equipped with Trimble NetR9 receivers, while a user station is equipped with a Septentrio PolRx5 receiver (Leuven, Belgium). Results discussed in this section are based on data collected from these four stations for ten days across three months (every seventh day) starting from day of year 190 of year 2017 with 30 s sampling interval. CUT0, which is Curtin University’s IGS reference station, is arbitrarily selected as the reference station for PPP-RTK network processing. For this small scale network, satellite positions were determined using broadcast navigation messages. Ground truths of station coordinates were computed using Geoscience Australia’s AUSPOS service. The estimable satellite clocks of multi-GNSS observables were aligned to respective reference observables using IGS-MGEX satellite DCB files.

For this small network, differential ionosphere delays were weighted as informed in Table 7 to strengthen the underlying model, while other parameter settings are as in Table 2. In addition to processing PPP-RTK user mode, user position was also determined using an in-the-loop mode, whereby the user position is assumed to be unknown and unlinked in time, is estimated together with other network parameters. The associated parameter settings for PPP-RTK user processing were identical to that of network processing above, except that user position was assumed to be unknown and unlinked in time. To get enough samples for generating reliable convergence curves, PPP-RTK user processing and in-the-loop user positioning were repeated every 40 min and across ten days resulting in 340 data windows. Unlike processing in previous sections, network and user processing started at the same epoch for a fair comparison between PPP-RTK user and in-the-loop user positioning performance.

**Figure 11:**
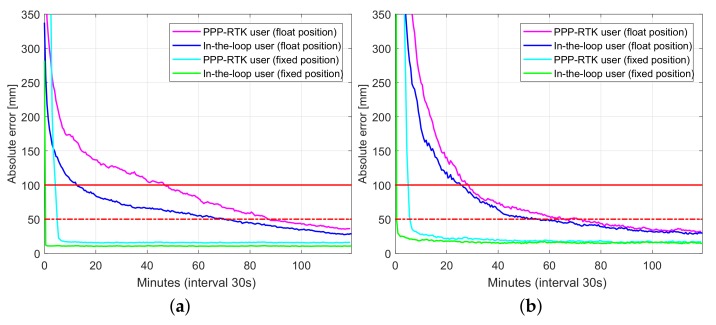
Convergence behavior (75% of samples) of UWA0 position errors based on processing of 430 data windows of GPS-only dual-frequency (L1-L2) small network data: PPP-RTK user vs. in-loop user. (**a**) Horizontal radial position error; (**b**) Up component error.

**Figure 12:**
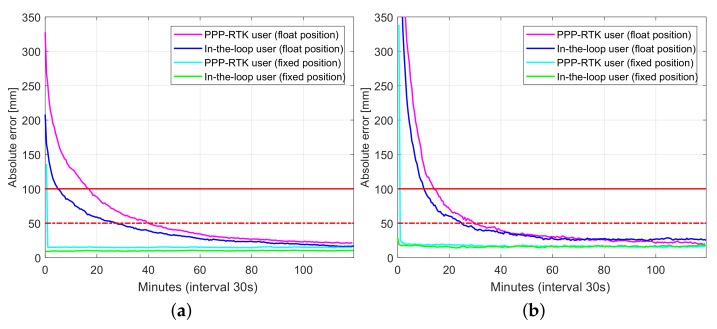
Convergence behavior (75% of samples) of UWA0 position errors based on processing of 430 data windows of multi-GNSS (GPS, BDS, Galileo), dual-frequency (L1-L2, B1-B2, E1-E5a) small network data: PPP-RTK user vs. in-loop user. (**a**) horizontal radial position error; (**b**) up component error.

Figure 11 and Figure 12 depict user convergence curves for GPS-only and triple system (GPS + BDS + Galileo) processing comparing performance of both PPP-RTK user and in-the-loop user mode processing, while Table 8 and Table 9 summarize corresponding convergence time for 1 dm and 5 cm (in brackets). Slightly better performance of in-the-loop user especially during the initialization is due to that in-the-loop user enjoys the use of full (variance-covariance) information, while a PPP-RTK user uses network products as deterministic corrections even at the initialization period. Hence, it is recommended to allow sometime (e.g., an hour) for network processing to converge before using its products. By using multi-GNSS, one can achieve almost instantaneous ambiguity resolved precise user position.

## 5. PPP-RTK Results: Low-Cost Single-Frequency Receivers

**Figure 13:**
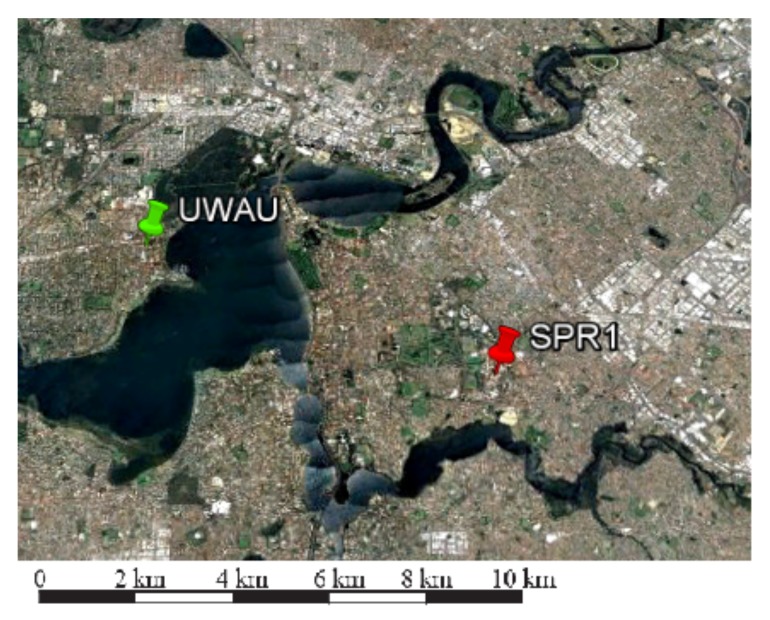
Multi-GNSS, single-frequency low-cost network (7 km) (Map data @ 2018 Google, Data SIO, NOAA, U.S. Navy. NGA. GEBCO) [26].

This section presents low-cost user positioning performance results of a single user (SPR1, shown in red in Figure 13) using single station (UWAU, shown in green in Figure 13). In this analysis, a real data campaign was set up with the reference station on top the Physics Building of University of Western Australia and user on top of Building 207 of Curtin University. Both stations are equipped with U-Blox M8 multi-GNSS receivers connected to U-Blox multi-GNSS patch antennas. Results discussed in the this section are based on multi-GNSS, single-frequency data collected from these two stations for three consecutive days (day of year 300, 301, and 302 of year 2017) with 30 s sampling interval. For this small scale network, satellite positions were determined using broadcast navigation messages. Ground truths of station coordinates were computed using batch processing of data form these stations and a nearby IGS reference station CUT0 (see Section 4). The estimable satellite clocks of multi-GNSS observables were aligned to respective reference observables using IGS-MGEX satellite DCB files.

Except parameter settings listed in Table 10, the parameter settings are as in Table 2. Similar to Section 4, user position was determined using both PPP-RTK user and in-the-loop-user modes, where differential ionosphere delays were weighted as informed in Table 10 strengthening underlying model. Large code noise standard deviation reflects the quality of code observations from low cost receivers connected to patch antennas. To get enough samples for generating reliable convergence curves, PPP-RTK user processing and in-the-loop user positioning were repeated every 10 min and across three days resulting in 356 data windows. Similar to Section 4, network and user processing started at the same epoch for a fair comparison between PPP-RTK user and in-the-loop user positioning performance.

**Figure 14:**
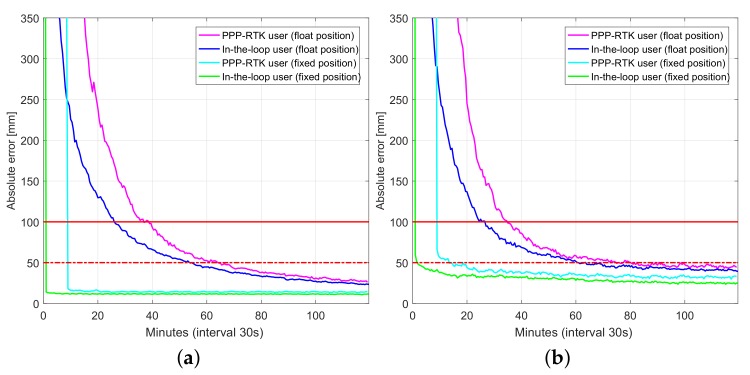
Convergence behavior (75% of samples) of SPR1 position errors based on processing of 356 data windows of multi-GNSS (GPS, BDS, Galileo), single-frequency (L1, B1, E1) low-cost network data: PPP-RTK user vs. in-loop user. (**a**) horizontal radial position error; (**b**) up component error.

Figure 14 depicts user convergence curves triple system (GPS+BDS+Galileo), single-frequency processing comparing performance of both the PPP-RTK user and in-the-loop user mode processing, while Table 11 summarizes corresponding convergence time for 1 dm and 5 cm (in brackets). Better performance of in-the-loop user especially during the initialization is due to that in-the-loop user enjoys the use of full (variance-covariance) information, while the PPP-RTK user uses network products as deterministic corrections even at the initialization period.

## 6. Conclusions

In this contribution, we provided numerical insights into the role taken by the multi-GNSS integration in delivering fast and high-precision positioning solutions using PPP-RTK. With the aid of Curtin PPP-RTK platform, data-sets of GPS, BDS and Galileo were processed in stand-alone and combined forms. Given the ground-truth coordinates of several single-receiver users, a large number of samples of the positioning errors (∼3800 samples) were collected so as to compute representative positioning convergence curves (cf. Figure 6, Figure 7, Figure 8 and Figure 9). In case of the Australia-wide GPS-only ambiguity-float setup, 90% of the horizontal positioning errors (kinematic mode) were shown to become less than five centimeters after 103 min. The stated required time is reduced to 66 min for the corresponding GPS + BDS + Galieo setup. The time is further reduced to 15 min by applying single-receiver ambiguity resolution. We also showed the impact of the latency in sending the network-derived corrections on the user positioning performance (cf. Figure 9). With a latency of 15 s, it takes around 15 min to have multi-GNSS ambiguity-fixed positioning errors less than 1 dm. For the corresponding ambiguity-float case, more time is required. In that case, the required time increases to 45 min.

We also presented multi-GNSS PPP-RTK results obtained by single-frequency low-cost receivers for which a ‘small-scale’ network is considered. The PPP-RTK user results were compared with the so-called ‘in-the-loop’ user, that is, the user’s data are simultaneously processed together with those of the network. While the PPP-RTK ambiguity-fixed positioning errors become less than 1 dm after 14 min, the corresponding in-the-loop counterparts only require 2 min to reach 1 dm.

## Figures and Tables

**Table 1:** Estimable GNSS (Global Navigation Satellite System) network-derived parameters formed by an S-basis [16]. The additional parameters $\Delta x_{1}\left(1\right)$ and $\tau_{1}\left(1\right)$ (within {.}) are taken as S-basis for the small-to-regional scale networks, i.e., when $c_{r}^{s}\approx c_{1}^{s}$ and $g_{r}^{s}\approx g_{1}^{s}$ (r=2,…,n). The argument (i) stands for the epoch index.

Position increments	$\Delta {\tilde{x}}_{r}\left(i\right)=\Delta x_{r}\left(i\right)-\left\{\Delta x_{1}\left(1\right)\right\}$
ZTDs (Zenith Tropospheric Delays)	${\tilde{\tau }}_{r}\left(i\right)=\tau_{r}\left(i\right)-\left\{\tau_{1}\left(1\right)\right\}$
Ionospheric delays	${\tilde{\iota }}_{r}^{s}\left(i\right)=\iota_{r}^{s}\left(i\right)+d_{r,GF}\left(1\right)-d_{,GF}^{s}\left(1\right)$
Satellite clocks	$d{\tilde{t}}^{s}\left(i\right)=\left(dt^{s}\left(i\right)+d_{,IF}^{s}\left(1\right)\right)-\left(dt_{1}\left(i\right)+d_{1,IF}\left(1\right)\right)-\left\{c_{1}^{sT}\Delta x_{1}\left(1\right)\right\}-\left\{g_{1}^{s}\tau_{1}\left(1\right)\right\}$
Receiver clocks	$d{\tilde{t}}_{r}\left(i\right)=dt_{r}\left(i\right)-dt_{1}\left(i\right)+d_{r,IF}\left(1\right)-d_{1,IF}\left(1\right)$
Sat. phase biases	${\tilde{\delta }}_{,j}^{s}\left(i\right)=\delta_{,j}^{s}\left(i\right)+\left(\mu_{j}\left[d_{,GF}^{s}\left(1\right)-d_{1,GF}\left(1\right)\right]-\left[d_{,IF}^{s}\left(1\right)-d_{1,IF}\left(1\right)\right]\right)-\delta_{1,j}\left(1\right)-\lambda_{j}z_{1,j}^{s}$
Rec. phase biases	${\tilde{\delta }}_{r,j}\left(i\right)=\delta_{r,j}\left(i\right)+\left(\mu_{j}\left[d_{r,GF}\left(1\right)-d_{1,GF}\left(1\right)\right]-\left[d_{r,IF}\left(1\right)-d_{1,IF}\left(1\right)\right]\right)-\delta_{1,j}\left(1\right)+\lambda_{j}\left(z_{r,j}^{1}-z_{1,j}^{1}\right)$
Sat. code biases	${\tilde{d}}_{,j}^{s}\left(i\right)=\left(d_{,j}^{s}\left(i\right)-\left[d_{,IF}^{s}\left(1\right)+\mu_{j}d_{,GF}^{s}\left(1\right)\right]\right)-\left(d_{1,j}\left(1\right)-\left[d_{1,IF}\left(1\right)+\mu_{j}d_{1,GF}\left(1\right)\right]\right)$
Rec. code biases	${\tilde{d}}_{r,j}\left(i\right)=d_{r,j}\left(i\right)-d_{r,j}\left(1\right)-\left(\left[d_{r,IF}\left(1\right)-d_{1,IF}\left(1\right)\right]+\mu_{j}\left[d_{r,GF}\left(1\right)-d_{1,GF}\left(1\right)\right]\right)$
Ambiguities	${\tilde{z}}_{r,j}^{s}=\left(z_{r,j}^{s}-z_{1,j}^{s}\right)-\left(z_{r,j}^{1}-z_{1,j}^{1}\right)$
S-basis parameters	$\left\{\Delta x_{1}\left(1\right)\right\},\;\left\{\tau_{1}\left(1\right)\right\},\;dt_{1}\left(i\right),d_{1,j}\left(1\right),\;\delta_{1,j}\left(1\right),\;d_{r\neq 1,j=1,2}\left(1\right),\;d_{,j=1,2}^{s}\left(1\right),\;z_{1,j}^{s},\;z_{r,j}^{1}$

${\left(.\right)}_{,IF}=\frac{1}{\mu_{2}-\mu_{1}}\left[\mu_{2}{\left(.\right)}_{,1}-\mu_{1}{\left(.\right)}_{,2}\right];\;{\left(.\right)}_{,GF}=\frac{1}{\mu_{2}-\mu_{1}}\left[{\left(.\right)}_{,2}-{\left(.\right)}_{,1}\right]$.

**Table 2:** Parameter settings for Australia-wide network processing.

**Stochastic Model with Sinusoidal Elevation Dependent Function**	
Zenith phase noise standard deviation	3 mm
Zenith code noise standard deviation	30 cm
Elevation weighting	Sine function
Elevation cut-off	10${}^{\circ }$
**Dynamic Model**	
Ambiguities	Time-constant
Satellite biases	Time-constant
Receiver biases	Time-constant
Troposphere delays (Random-walk)	$q_{t}=0.0001\;m/\sqrt{s}$
Ionosphere delays	Unlinked in time
Satellite clocks	Unlinked in time
Receiver clocks	Unlinked in time
**Ambiguity Resolution**	
Ambiguity resolution	Partial fixing
Expected minimum success rate ($P_{0}$)	0.999
Fixing BDS Geostationary satellite ambiguities	No
**Ionosphere**	
Ionosphere spatial model	None (Ionosphere float)

**Table 3:** (50%) Convergence time of user estimates to 1 dm (in brackets 5 cm) with respect to ground truth.

System	Fixed (min)		Float (min)	
	Horizontal	Up	Horizontal	Up
GPS	10.0 (16.0)	10.0 (16.5)	11.0 (25.0)	10.5 (22.5)
GPS + BDS	9.5 (12.5)	9.5 (13.0)	10.0 (21.5)	9.5 (20.5)
GPS + Galileo	9.0 (12.5)	9.0 (10.5)	10.0 (21.5)	9.5 (20.5)
GPS + BDS + Galileo	7.5 (10.0)	8.0 (10.5)	8.5 (19.0)	8.5 (18.5)

**Table 4:** (75%) Convergence time of user estimates to 1 dm (in brackets 5 cm) with respect to ground truth.

System	Fixed (min)		Float (min)	
	Horizontal	Up	Horizontal	Up
GPS	16.5 (20.5)	17.0 (23.5)	23.0 (53.5)	21.5 (57.5)
GPS + BDS	13.5 (15.0)	14.0 (20.0)	18.5 (42.0)	19.5 (52.5)
GPS + Galileo	13.0 (16.0)	13.5 (17.5)	18.5 (42.0)	18.5 (49.5)
GPS + BDS + Galileo	10.5 (12.0)	11.0 (14.0)	16.0 (36.5)	17.5 (46.0)

**Table 5:** (90%) Convergence time of user estimates to 1 dm (in brackets 5 cm) with respect to ground truth.

System	Fixed (min)		Float (min)	
	Horizontal	Up	Horizontal	Up
GPS	23.5 (28.0)	26.5 (49.5)	47.5 (102.5)	47.5 (102.5)
GPS + BDS	18.5 (20.0)	21.5 (59.5)	36.0 (81.0)	40.5 (100.5)
GPS + Galileo	17.5 (20.0)	18.5 (35.0)	35.0 (85.0)	37.0 (91.5)
GPS + BDS + Galileo	14.0 (15.0)	15.5 (44.5)	30.0 (65.5)	33.5 (89.5)

**Table 6:** Parameter settings for Australia-wide network processing with satellite clock model; Other parameter settings are as in Table 2.

Dynamic Model	
Satellite clocks	$q_{c}$ = 0.001 m/$\sqrt{s}$

**Table 7:** Parameter settings for small network processing; Other parameter settings are as in Table 2.

Ionosphere	
Ionosphere spatial model	Ionosphere weighted
Applicable inter-station distance ($l_{0}$)	2 km
Standard deviation of undifference ionosphere observables	0.01 m/$l_{0}$

**Table 8:** (75%) Convergence time of user coordinate estimates to 1 dm (in brackets 5 cm) using GPS-only data of the small-scale network (ionosphere-weighted model).

Method	Fixed (min)		Float (min)	
	Horizontal	Up	Horizontal	Up
PPP-RTK user	4.5 (5.0)	5.0 (5.5)	47.0 (87.0)	28.5 (64.5)
In-the-loop user	0.5 (0.5)	0.5 (0.5)	12.5 (70.0)	25.5 (55.5)

**Table 9:** (75%) Convergence time of user coordinate estimates to 1 dm (in brackets 5 cm) using multi-GNSS data of the small-scale network (ionosphere-weighted model).

Method	Fixed (min)		Float (min)	
	Horizontal	Up	Horizontal	Up
PPP-RTK user	1.0 (1.0)	0.5 (0.5)	17.0 (40.5)	14.0 (30.0)
In-the-loop user	0.0 (0.0)	0.0 (0.0)	5.0 (27.5)	10.5 (25.0)

**Table 10:** Parameter settings for low-cost network processing; other parameters are as in Table 2.

**Stochastic Model with Sinusoidal Elevation Dependent Function**	
Zenith phase noise standard deviation	GPS: 3 mm, BDS: 4 mm, Galileo: 3 mm
Zenith code noise standard deviation	GPS: 60 cm, BDS: 80 cm, Galileo: 60 cm
**Dynamic Model**	
Receiver biases	Unlinked in time
**Ionosphere**	
Ionosphere spatial model	Ionosphere weighted
Applicable inter-station distance ($l_{0}$)	2 km
Standard deviation of undifference ionosphere observables	0.1 m/$l_{0}$

**Table 11:** (75%) Convergence time of user coordinate estimates to 1 dm (in brackets 5 cm) using low-cost multi-GNSS receivers (ionosphere-weighted model).

Method	Fixed (min)		Float (min)	
	Horizontal	Up	Horizontal	Up
PPP-RTK user	9.0 (9.0)	9.0 (13.5)	39.0 (64.5)	35.5 (75.0)
In-the-loop user	1.0 (1.0)	1.0 (2.0)	26.0 (54.0)	27.0 (61.0)
